# Diagnosis of hepatic alveolar echinococcosis:18F-FDG-PET activity compared to the Echinococcus multilocularis Ulm Ultrasound Classification

**DOI:** 10.1055/a-2744-4139

**Published:** 2025-12-01

**Authors:** Wolfgang Kratzer, Sibylle Steinkellner, Dennis Skotnik, Lynn Peters, Beate Gruener, Nina Eberhardt

**Affiliations:** 1 27197 Department of Internal Medicine I, Ulm University Hospital, Ulm, Germany; 2 27197 Department of Internal Medicine III, Division of Infectious Diseases, Ulm University Hospital, Ulm, Germany; 3 27197 Department of Nuclear Medicine, Ulm University Hospital, Ulm, Germany

**Keywords:** hepatic alveolar echinococcosis, 18F-Fluorodeoxyglucose-Positron-Emission-Tomography, 18F-FDG-PET/CT, ultrasound, classification scheme, EMUC-US

## Abstract

**Aim:**

Alveolar echinococcosis (AE) is a rare, potentially fatal zoonosis with highly heterogeneous morphology. This study compares AE lesions in B-scan ultrasound, categorized according to the Echinococcus multilocularis Ulm Classification – Ultrasound (EMUC-US), with the maximum standardized uptake value (SUVmax) in 18F-Fluorodeoxyglucose Positron-Emission-Tomography (18F-FDG-PET/CT). 18F-FDG-PET/CT is the gold standard for evaluating disease activity, with SUVmax as the key parameter indirectly reflecting AE lesion activity.

**Methods:**

Retrospective analysis of data from the German National Echinococcosis Database. A total of 121 patients with 18F-FDG-PET/CT and B-scan ultrasound (US) between 2018–2019 were included. Based on EMUC-US, AE liver lesions were compared with the corresponding SUVmax in PET/CT. Additionally, SUV ratios (SUVTLR=tumor SUVmax/liver SUVmean) were calculated.

**Results:**

The mean SUVmax, regardless of the EMUC-US subtype, was 6.0 ± 3.3 (range: 2.4–18.0). SUVmax comparison between subtypes shows significant differences (p<0.001). The highest SUVmax and SUVTLR were measured for the pseudocystic pattern with a mean of 9.2 ± 3.5 (range: 4.1–18.0). In contrast, the metastasis-like pattern yielded 3.7 ± 0.9 (range: 2.4–5.8) and the lowest SUVTLR. An SUVmax of 6.1 ± 3.3 (range: 2.6–16.8) was measured for the hailstorm pattern and 5.8 ± 2.2 (range: 3.6–10.4) for the hemangioma-like pattern.

**Conclusion:**

The results show significant differences between specific US patterns and the corresponding SUVmax. Lesions with very high or low SUV correlate with characteristic morphological patterns. Hence, in clinical practice B-scan can be a valuable bedside tool for assessing certain lesions. For evaluating inflammatory activity, 18F-FDG-PET/CT remains the method of choice.

## Introduction

Alveolar echinococcosis (AE) is a rare, potentially fatal zoonosis 1 . In recent years, a worldwide expansion has been observed 2 . With a 5–20-year asymptomatic latency, AE usually manifests as a hepatic tumour in human accidental intermediate hosts. The different forms of manifestation have high potential for differential diagnosis and often lead to lengthy diagnostic processes 3 4 .

Surgical resection is the preferred therapy in operable cases, whereas advanced, inoperable stages are treated with long-term anthelmintic therapy using benzimidazoles (BMZ) 5 . Imaging diagnostics of AE have gained importance in recent years 6 7 8 . The development of AE classification schemes for magnetic resonance imaging (MRI), B-scan ultrasonography (US) and computed tomography (CT) has significantly improved diagnosis, pathophysiological understanding and comparability of AE findings 9 10 11 . Yet they remain underrepresented and infrequently applied in research literature. A classification of AE for 18F-Fluorodeoxyglucose Positron-Emission-Tomography (18F-FDG-PET/CT) does not yet exist.

Since the first description of AE in 18F-FDG-PET/CT by Reuter et al. (1999), positron emission tomography (PET) remains the only method for assessing the metabolic inflammatory activity of AE lesions 12 . However, it should be noted that AE lesions themselves do not demonstrate 18F-FDG uptake; the observed signal arises from perilesional inflammation – an expression of the inflammatory immune response around the parasitic lesion 12 13 14 . Data from Stumpe et al. emphasize that increased FDG uptake does not correspond to the vitality of the AE lesion or the parasite 14 . The maximum standardized uptake value (SUVmax), a key parameter in PET, measuring the highest tracer uptake in a lesion, is therefore only an indirect marker of parasite viability 13 . The histology of the lesion and its close proximity to the hostʼs inflammatory cells also explains the central role of the patientʼs immunocompetence in AE. In immunodeficient patients, hepatic AE was usually diagnosed in earlier stages, often as an incidental finding. Immunocompromised patients showed lesions with atypical imaging more often and exhibited a lower sensitivity to serological tests 15 . In this population imaging becomes even more important for the diagnosis of AE 13 . Comparative studies of 18F-FDG-PET/CT, B-scan and contrast-enhanced ultrasound (CEUS) showed a correlation between metabolic activity and vascularization, as well as a better visualization of lesion size in CEUS 16 . The correlation between 18F-FDG-PET/CT viability and immunological parameters was reconfirmed by Husmann et al. in a recent study 17 . Combining 18F-FDG-PET/CT and serological markers for therapy monitoring appears to be superior to BMZ therapy controlled by PET/CT alone 18 . Long-term follow-up studies have shown a heterogeneous response of hepatic AE lesions in 18F-FDG-PET/CT to BMZ, which makes follow-up necessary even years after diagnosis 19 20 .

Due to the limited availability of PET scanners and the radiation exposure to patients, numerous studies have been initiated to evaluate the efficiency of other imaging modalities to compare these findings with the 18F-FDG-PET activity 16 21 22 23 . To date, there are no studies comparing hepatic AE lesions classified by B-scan ultrasound and the activity in 18F-FDG-PET/CT. The aim of this study was to compare the sonomorphologic presentation of AE lesions, classified according to EMUC-US, and the 18F-FDG-PET/CT activity.

## Material and Methods

A retrospective analysis was conducted using data from the German National Echinococcosis Database 24 . “Confirmed”, “probable” and “possible” AE cases according to the WHO case definition were included in the database 25 . This study was approved by the local ethics committee and conducted in accordance with the Declaration of Helsinki (ref. no. 166/13). The image data used are part of the database. Upon inclusion, the patients had given their written consent for the use of the collected data for future retrospective data analyses. The data was analysed anonymously. All patients were ≥18 years old at the time of inclusion in the database.

### Patients

**Fig. 1:**
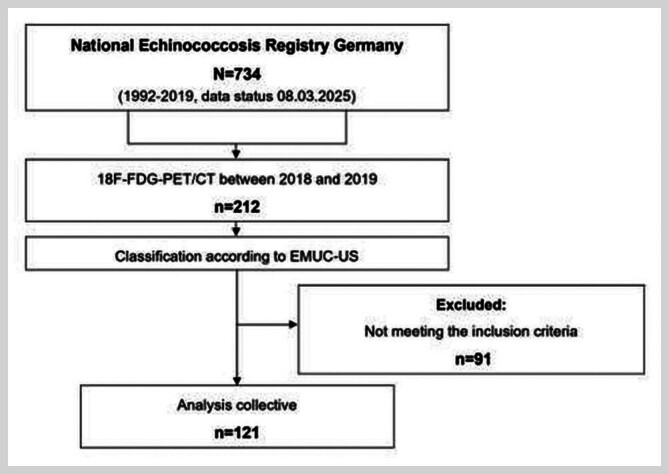
Flowchart showing case number generation based on AE cases in the German National Echinococcosis Database (1992–2019) (n=734, as of data evaluation 08.03.2025). (AE=alveolar echinococcosis; n=sample size; WHO=World Health Organisation; EMUC=Echinococcus multilocularis Ulm Classification; US=Ultrasound; PET=positron emission tomography).

From the data pool of the German National Echinococcosis Database (n=734; Data status 08.03.2025), 121 patients (71 women, 50 men) were included in the study ( Fig. 1 ).

Inclusion criteria included an 18F-FDG-PET/CT between 2018 and 2019 at our hospital as well as an abdominal US examination with a classification of B-scan sonomorphology according to the EMUC-US 7 . The presence of a liver lesion was obligate, patients with solely extrahepatic AE manifestation were excluded. AE cases considered “confirmed” or “probable” (WHO case definition) were evaluated. “Possible” cases were excluded 25 . This approach reflects clinical realitiy, reduces selection bias, and follows WHO recommendations regarding the relevance of probable cases 25 26 .

Of 211 eligible cases 90 more were excluded for following reasons:

- R0 liver resection before imaging (n=53)
- Missing in-house US examination (n=22)
- Time span greater than 12 months between US and 18F-FDG-PET/CT (n=9)
- Unclassifiable AE lesion according to EMUC-US (n=3)
- EMUC-US ossification-pattern as the predominant lesion type (n=2)
- Patient with secondary cholangitis and abscess formation, potentially confounding FDG-uptake (n=1)

### US examinations and classification according to EMUC-US

The sonographic findings were collected at the Ultrasound Department at University Hospital Ulm between 2018 and 2019 by residents and specialists in internal medicine and radiology with in-depth training in US during clinical routine exams. Abdominal examinations were performed with convex probes (C5–1 MHz, C6–1 MHz).

**Fig. 2:**
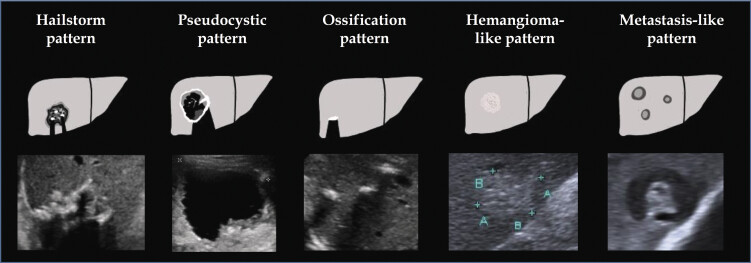
Overview and schematic representation of the EMUC-US classification system according to Kratzer et al. 10 . Author’s illustration from Philipp J, Schmidberger J, Schlingeloff P et al. Differentiation of hepatic alveolar echinococcosis with a hemangioma-like pattern compared to typical liver hemangioma using contrast-enhanced ultrasound: a pilot study. Infection 2023; 51: 159–168. DOI: https://doi.org/10.1007/s15010–022–01866-z ; Copyright 2023 by the authors. This article is an open access article distributed under the terms and conditions of the Creative Commons Attribution (CC BY) license ( https://creativecommons.org/licenses/by/4.0/ ). No changes were made to the original illustration. (AE=alveolar echinococcosis; EMUC=Echinococcus multilocularis Ulm classification; US=ultrasound); the ultrasound images shown are from the current patient collective and are taken from the Echinococcosis Database Germany.).

According to EMUC-US, AE liver lesions were categorized into five subtypes ( Fig. 2 ) 10 :

1. Hailstorm pattern
2. Pseudocystic pattern
3. Hemangioma-like pattern
4. Metastasis-like pattern
5. Ossification pattern

Furthermore, the following parameters were collected: number of lesions, localisation based on the liver segments, longest diameter of the largest lesion. If the sonomorphology of the reference lesion showed characteristic features of different EMUC-US patterns, it was assigned to the predominant type, i.e. the pattern of the largest lesion. If it was not possible to assign the lesion to a pattern of EMUC-US, it was documented as “unclassifiable”. Due to an insufficient number of cases, lesions of the ossification pattern were not included in the analysis (n=2).

As part of the study, a second reading was conducted, with two independent, experienced examiners (5 and >10 years of experience in AE ultrasound) performing secondary review and categorization of the sonographic findings according to EMUC-US. Additionally, US examinations before and after the selected 18F-FDG-PET/CT were assessed for pattern changes. Cases where a pattern change was observed in close temporal proximity to the PET/CT were excluded. All US findings were supervised by a Senior Consultant with over 20 years of professional experience in US imaging to ensure consistency and reliability.

### 18F-FDG-PET/CT examinations and SUV measurements

The 18F-FDG-PET/CT included in the study were conducted between 2018 and 2019 at University Hospital Ulm as part of clinical routine examinations. The Biograph mCT-S scanner (Siemens Healthineers, Erlangen, Germany) was used, allowing full 3D imaging with time of flight (TOF) and point spread function corrections. All patients fasted for at least six hours prior to the examination, and a dose of 320–340 MBq of 18F-FDG was administered intravenously. After an uptake time of 60–90 minutes, during which patients remained at rest, imaging was performed. PET measurements at each bed position lasted 2.5 minutes. CT scans were performed during shallow expiration, mostly with intravenous contrast (Ultravist-300), acquired at a slice thickness of 1.5–5.0 mm, with reconstruction intervals of the same or smaller thickness. Quantitative PET images were evaluated at a slice thickness of 5.0 mm and a pixel size of 4.07 mm.

**Fig. 3:**
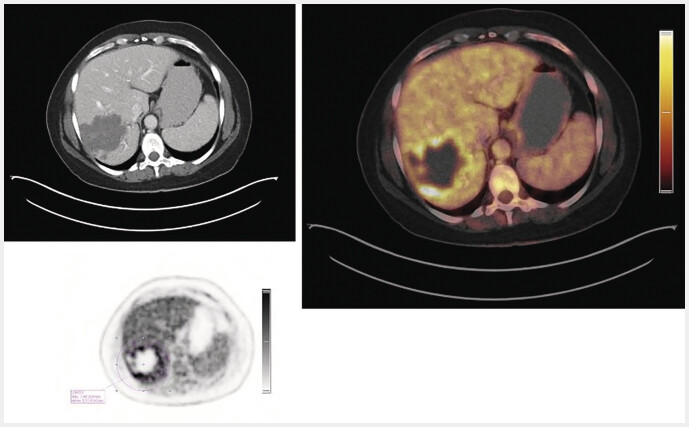
18F-FDG-PET/CT examination and SUV measurement of an active AE lesion in a 52-year-old, non-liver resected female patient with the respective color scale. (Right) Fusion PET/CT imaging. (Left, above) CT imaging (Left, down) Quantitative measurement of SUVmax (7.5) in the PET examination for a specific VOI (circle). (AE=alveolar echinococcosis; CT=computed tomography; 18F-FDG-PET=18F-fluorodeoxyglucose positron emission tomography; SUVmax=maximum standardised uptake value; VOI=volume of interest); The image data shown are from the current patient collective from the Echinococcosis Database Germany.

PET and CT images were fused using a Siemens Syngo Via workstation (VB60) for alignment at the same slice thickness in the soft tissue window. SUV values (SUVmax and SUVmean) were measured by an experienced nuclear medicine physician (>5 years PET experience). PET-positive lesions, defined by visually increased 18F-FDG uptake compared to surrounding liver parenchyma, were measured using VOI (volume of interest) tools, which automatically contoured areas with elevated uptake ( Fig. 3 ). SUVmean values of normal liver parenchyma were measured in representative areas without vascular structures for comparison. PET-negative lesions were defined as lesions without visible contrast from the background liver tissue. For PET negative lesions SUV measurements were conducted in peripheral regions of these lesions. Additionally, SUV ratios were calculated, comparing lesion SUVmax to the SUVmean of the liver backround, following the tumor-to-liver ratio (SUV _TLR_ ) methodology used in oncology.

### Statistics

The statistical analysis was performed using Microsoft Excel (Microsoft 365) and the data analysis programme SPSS (Version 30.0, IBM). Descriptive analyses included absolute/relative frequencies, means, measures of central tendency/dispersion, visualized in box plots. The Shapiro-Wilk test was used to assess normal distribution. For non-normally distributed interval data, the Mann-Whitney U test was used. Bonferroni correction adjusted significance levels for multiple pairwise comparisons. Possible influencing factors were assessed via bivariate correlations (Pearson or Spearman, depending on data distribution). Differences in frequency distributions of categorical or dichotomous variables were analyzed using the Chi² test or Fisherʼs Exact Test when applicable. Comparisons across more than two independent groups were performed using the Kruskal-Wallis test with Eta² as effect size (large effect: Eta² > 0.14). A two-sided p-value < 0.05 (α = 0.05) was considered statistically significant.

## Results

A total of 121 patients [58.7% (n=71) women; 41.3% (n=50) men, mean age 60.2 ± 15.9 years; range: 21–85] were included. Based on the AE WHO case definition, 61.2% (n=74) of patients were classified as “probable” and 38.8% (n=47) as “confirmed”. The average interval between the US examination and the 18F-FDG-PET/CT was 4.4 ± 3.4 months (range: 0–12). During the study period, 84.3% (n=102) of the patients received BMZ therapy (mean duration in total 43.2 ± 61.9 months (range: 0–297).

### Sonographic findings

The mean number of AE lesions detected in the B-scan was 2.6 ± 2.7 (range: 1–20). In 31.4% (38/121) the right lobe, in 15.7% (19/121) the left lobe and in 52.9% (64/121) both liver lobes were affected. AE lesions were most frequently detected in liver segments VII (47.9% (n=58/121), VIII (47.1%, n=57/121) and V (47.1%, n=57/121). The average size of the *E. multilocularis* lesions was 64 ± 38 mm (range: 9–194).

### Classification according to EMUC-US

Based on the US examination, the liver lesions were categorised according to EMUC-US 11 . At 45.5% (55/121), most lesions corresponded to hailstorm pattern. The pseudocystic pattern accounted for 17.4% (21/121), the hemangioma-like pattern for 14.9% (18/121) and the metastasis-like pattern for 22.3% (27/121) ( Table 1 ). An overall comparison of the different EMUC-US patterns showed significant differences in lesion size (p<0.001; H=53.61). Lesions of the pseudocystic pattern had the longest diameters at 106 mm (range: 41–194). In comparison, lesions that were assigned to the metastasis-like pattern were on average only 27 mm (range: 9–84) in length ( Table 1 ). Lesions of the ossification pattern were not part of the data analysis due to an insufficient number of cases.

**Table:** **Table 1** Frequency distribution and size of the liver lesion according to the EMUC-US pattern (n=121; Kruskal–Wallis test: p<0.001; H=53.61, Eta²=0.432).

EMUC-US pattern	n (%)	Mean lesion size (mm) ± SD Median (Min-Max)
Hailstorm pattern	55 (45.4%)	63.8 ± 27,0 64.0 (23.0–117.0)
Metastasis-like pattern	27 (22.3%)	26.7 ± 20.4 20.0 (9.0–84.0)
Pseudocystic pattern	21 (17.4%)	106.4 ± 37.7 105.0 (41.0–194.0)
Hemangioma-like pattern	18 (14.9%)	67.4 ± 29.6 63.0 (10.0–132.0)
Abbreviations: EMUC=Echinococcus multilocularis Ulm Classification; US=Ultrasound; mm=Millimetre; SD=Standard deviation; Min=Minimum; Max=Maximum; n=sample size; H=Kruskal-Wallis H statistic.		

### SUVmax values and SUV _TLR_ according to the EMUC-US pattern

Mean SUVmax across EMUC-US subtypes was 6.0 ± 3.3 (range: 2.4–18.0). The overall comparison of SUVmax between the different US subtypes shows significant differences (p=<0.001, H=40.26). The highest SUVmax were measured for the pseudocystic pattern with a mean value of 9.2 ± 3.5 (range: 4.1–18.0).

In comparison, SUVmax for liver lesions of the metastasis-like pattern only yielded 3.7 ± 0.9 (range: 2.4–5.8). For the hailstrom pattern, an average SUVmax of 6.1 ± 3.3 (range: 2.6–16.8) was measured, and for the hemangioma-like pattern an average SUVmax of 5.8 ± 2.2 (range 3.6–10.4) ( Table 2 ).

**Table:** **Table 2** Measures of central tendency and dispersion of SUVmax values according to the EMUC-US pattern (n=121; Kruskal–Wallis test: p<0.001; H=53.61, Eta²=0.318).

EMUC-US pattern	N (%)	SUVmax Mean ± SD Median (Min.-Max.)
Hailstorm pattern	55 (45.45%)	6.1 ± 3.3 4.8 (2.6–16.8)
Metastasis-like pattern	27 (22.31%)	3.7 ± 0.9 3.7 (2.4–5.8)
Pseudocystic pattern	21 (17.36%)	9.2 ± 3.5 8.5 (4.1–18.0)
Hemangioma-like pattern	18 (14.88%)	5.8 ± 2.2 4.8 (3.6–10.4)
Abbreviations: SUVmax=maximum standardized uptake value; EMUC=Echinococcus multilocularis Ulm Classification; US=Ultrasound; mm=Millimetre; SD=Standard deviation; Min=Minimum; Max=Maximum, n=sample size; H=Kruskal-Wallis H statistic.		

Pairwise comparisons demonstrated significant differences in SUVmax distribution between the EMUC-US-patterns ( Table 3 ). Specifically, the metastasis-like pattern showed significantly lower SUVmax compared to the other patterns. The metastasis-like pattern showed significantly lower SUVmax compared to the hailstorm pattern (3.7 ± 0.9 vs. 6.1 ± 3.3; p=0,001). Significant SUVmax rank differences were also noted between the metastasis-like and the pseudocystic subtype (3.7 ± 0.9 vs. 9.2 ± 3.5; p=<0.001). Moreover, a significant divergence in SUVmax was noted between and the metastasis-like pattern and the hemangioma-like pattern (3.7 ± 0.9 vs. 5.8 ± 2.2; p=0.004).

**Table:** **Table 3** Results of the Dunn-Bonferroni test as a post-hoc analysis following a Kruskal-Wallis test. Pairwise comparisons of SUVmax between the individual EMUC-US patterns; p-values correspond to adjusted significance levels after Bonferroni correction; statistically significant values after Bonferroni correction are highlighted in bold (n=121).

EMUC-US pattern Mean ± SD Median (Min-Max)	Hailstorm pattern	Pseudocystic pattern	Hemangioma-like pattern	Metastasis-like pattern
**Hailstorm pattern** 6.1 ± 3.3 4.8 (2.6–16.8)	–	0.002	1.000	0.001
**Pseudocystic pattern** 9.2 ± 3.5 8.5 (4.1–18.0)	0.002	–	0.074	<0.001
**Hemangioma-like pattern** 5.8 ± 2.2 4.8 (3.6–10.4)	1.000	0.074	–	0.004
**Metastasis-like pattern** 3.7 ± 0.9 3.7 (2.4–5.8)	0.001	<0.001	0.004	–
Abbreviations: SUVmax=maximum standardized uptake value; EMUC=Echinococcus multilocularis Ulm Classification; US=Ultrasound; SD=Standard deviation; Min=Minimum; Max=Maximum, n=sample size.				

Additionally, when comparing the hailstorm lesion type to the pseudocystic pattern, statistically significant rank differences in SUVmax were observed (6.1 ± 3.3 vs. 9.2 ± 3.5; p=0.002). No statistically significant differences were found in SUVmax rank comparisons between the hemangioma-like and the pseudocystic, or the hailstorm and the hemangioma-like pattern.

To contextualize the SUVmax, SUV ratios (SUV _TLR_ ) were calculated by dividing the SUVmax of the AE lesion by the SUVmean of the liver background for each EMUC-US pattern. The analysis revealed significant differences for SUV _TLR_ across the four subtypes (p < 0.001, Eta² = 0.349). Post-hoc tests confirmed significant differences between most patterns, except between the hailstorm and hemangioma-like pattern ( Table 4 ). Metastasis-like lesions exhibited the lowest SUV _TLR_ (1.5 ± 0.3), whereas pseudocystic lesions showed the highest SUV _TLR_ (4.1 ± 1.7).

**Table:** **Table 4** SUV ratio (SUV _TLR_ ) between the SUVmax of AE lesions and the SUVmean of the liver background, categorized by EMUC-US pattern. (n=121; Kruskal-Wallis-Test, H=43,81, p=<0,001, Eta ^2^ =0,349).

	SUVmax AE lesion	SUVmean liver backround	SUV _TLR_
EMUC-US pattern	Mean ± SD Median (Min.-Max.)	Mean ± SD Median (Min.-Max.)	Mean (± SD)
Hailstorm pattern	6.1 ± 3.3 4.8 (2.6–16.8)	2.3 ± 0.3 2.3 (1.8–3.2)	2.6 (± 1.5)
Metastasis-like pattern	3.7 ± 0.9 3.7 (2.4–5.8)	2.4 ± 0.4 2.3 (1.7–3.7)	1.5 (± 0.3)
Pseudocystic pattern	9.2 ± 3.5 8.5 (4.1–18.0)	2.3 ± 0.3 2.3 (1.6–2.8)	4.1 (± 1.7)
Hemangioma-like pattern	5.8 ± 2.2 4.8 (3.6–10.4)	2.4 ± 0.4 2.5 (1.5–3.0)	2.4 (± 0.9)
Abbreviations: AE=alveolar echinococcosis; SUVmax=maximum standardized uptake value; SUVmean=mean standardized uptake value; SUV _TLR_ =standardized uptake value – tumor to liver ratio; EMUC=Echinococcus multilocularis Ulm Classification; US=Ultrasound; SD=Standard deviation; Min=Minimum; Max=Maximum; n=sample size; H=Kruskal-Wallis H statistic.			

A correlation analysis revealed a significant correlation between lesion size and SUVmax, where larger lesions were associated with higher SUVmax. No other statistically significant correlations were identified.

## Discussion

This is the first study that compares the US morphology of AE liver lesions categorised according to EMUC-US with the metabolic activity in 18F-FDG-PET/CT and thus sheds light on the diagnostic value of both imaging methods 10 19 .

The results show a clear difference between the sonographic patterns according to EMUC-US and the SUVmax in 18F-FDG-PET/CT. The pseudodocytic pattern shows the highest SUVmax and SUV _TLR_ , whereas the metastasis-like pattern shows significantly lower SUVmax, along with the lowest SUV _TLR_ in comparison. At first glance, it may seem counterintuitive that the pseudocystic pattern shows the highest SUVmax. The actual immunological interaction between parasite and liver tissue occurs in this border zone. This area is marked by dense infiltration of macrophages and lymphocytes, fibrosis, neovascularization, and consequently increased metabolic activity, which results in higher FDG uptake. Thus, the elevated SUVmax is not due to the necrotic core but to the metabolically active peripheral rim 27 28 29 .

To date, 18F-FDG-PET/CT is the only established imaging method for determining metabolic activity and thus indirectly indicates the activity of the parasite through the bodyʼs own immune reaction 12 .

In recent years, several comparative studies have been conducted to evaluate the diagnostic efficiency of different imaging modalities for AE, comparing them with PET findings. When evaluating results based on classification schemes, studies show differences in SUVmax interpretation and vascularization patterns 16 21 22 23 .

A strong correlation was found for the comparison between 18F-FDG uptake in PET/CT and MRI findings using the Kodama classification. Azizi et al. revealed significant SUVmax differences between Kodama types 1–3 (non-microcystic) and type 4 and type 5 lesions (microcystic). All Kodama type 1, 90.9% of type 2 and 87.5% of type 3 showed increased FDG uptake in PET/CT. In contrast, all non-microcystic lesions (Kodama type 4 and 5) showed no abnormally increased FDG uptake. The results of Azizi et al. suggest a correlation between the presence of microcysts and increased metabolic activity in 18F-FDG-PET/CT. The absence of microcysts correlated strongly with a metabolically inactive disease 23 . Our study suggests a possible overlap between the EMUC-US metastasis-like pattern and Type 4 and 5 of the Kodama classification, all of which are characterized by low SUVmax.

Brumpt et al. demonstrated increased SUV in 18F-FDG-PET/CT for AE liver lesions with detectable microcalcifications on CT 21 , but this study did not include a corresponding classification scheme in the data analysis 11 . However, in a study using the classification scheme EMUC-CT (Echinococcus multilocularis Ulm Classification – computed tomography), this finding couldn’t be clearly confirmed 22 . CEUS appears to be of great importance in the future, although few comparative studies with 18F-FDG-PET are currently available 30 31 32 . In a study conducted by Kaltenbach et al. all 18F-FDG-PET-negative lesions showed no vascularisation on CEUS, which suggests that vascularisation in CEUS could be related to increased SUV in 18F-FDG-PET 33 .

One disadvantage of 18F-FDG PET/CT is the considerable radiation exposure. PET/MRI is a possible alternative, imaging method, but is currently only available at large hospitals in limited capacity 34 . In 2019, Lötsch et al. showed in a case study with four AE patients comparable diagnostic information for 18F-FDG-PET/MRI in AE management compared to 18F-FDG-PET/CT with a significantly reduced radiation exposure 35 . While the effective radiation dose for a whole-body 18F-FDG-PET/CT was 30.4–31.0 mSV, this could be reduced to the exposure of 18F-FDG alone [4.9–5.5 mSv) for PET/MRI in the study of Lötsch et al. 35 . Eberhardt et al. were able to confirm these results in a recent study 36 .

The metabolic activity of AE lesions is of clinical relevance, as it plays a key role, alongside serological findings, in determining the therapeutic approach. In summary, lesions of the EMUC-US pseudocystic pattern, as shown in our study, as well as Kodama type 1 and 2 show the highest SUVmax according to the current state of research and are considered to be the most metabolically active AE lesions 10 23 . The metastasis-like pattern according to EMUC-US shows the least or often no activity in 18F-FDG-PET/CT. In a 2022 retrospective study, Peters et al. were also able to show a correlation between morphology and clinical outcome: pseudocystic lesions were more often progressive, while metastatic lesions tended to remain stable often without the need for therapy 37 .

This underscores the importance of assessing lesion activity for tailoring treatment strategies. This information could be instrumental in optimizing patient care, particularly in resource-limited settings or for early-stage cases where the need for intervention remains uncertain. Whether lesions of the metastasis-like pattern thus correspond to an early stage or represent lesions that can be controlled and potentially contained by the hostʼs own immune system are currently subject to scientific research. The extent to which metabolic activity can be interpreted as a measure of a possible development cycle cannot currently be answered based on the available literature 38 . For a possible watch and wait strategy, this would be important diagnostic information relevant to treatment.

### Limitations

This study has several limitations. First, the small sample size reflects the rare nature of AE and the limited patient cohort available. Despite using the national Echinococcosis Database Germany to centralize AE cases, only 121 patients met the inclusion criteria due to dropouts. This led to unequal distribution among the five EMUC-US patterns, with the ossification pattern excluded due to insufficient cases (n=2). Future studies, ideally prospective, with larger cohorts, longer observation periods, and a multicenter approach would be desirable to address this limitation adequately.

Second, although ethically justifiable, the retrospective design resulted in time intervals of up to 12 months between US and 18F-FDG-PET/CT. Ideally, both exams should be performed on the same day or during the same hospital stay, with follow-up imaging incorporated into the analysis. Furthermore, the generally applicable limitations for ultrasound diagnostics (examination conditions, meteorism, obesity), as well as the examinerʼs experience, remain as limiting factors.

Third, we did not perform delayed PET acquisitions. Although increased sensitivity has been reported in selected cases, this has not been confirmed in larger multicenter studies 6 13 . As current international guidelines do not mandate delayed imaging, we considered the standard 1-h acquisition sufficient, while acknowledging that its potential added value warrants further investigation 39 40 .

## Conclusion

Although US is invaluable for morphological classification, it currently cannot replace 18F-FDG-PET/CT in the clinical follow-up routine, as metabolic activity often decreases more rapidly than morphological changes become apparent. 18F-FDG-PET/CT therefore remains the gold standard for evaluating disease activity and a key element in the AE long-term follow-up-strategy. However, the results of our study provide important diagnostic insights in terms of lesion activity especially useful at the time of initial diagnosis. This can be helpful to estimate how urgently patients need immediate therapy or referral to a specialized center, particularly in rural areas with limited resources.

### Statement of Ethics

The study was approved by the local ethics committee of the University and conducted in accordance with the Declaration of Helsinki (ref. no. 166/13). All data were analyzed anonymously.

### Data Availability Statement

The datasets used and analyzed during the current study are available from the corresponding author on reasonable request.

## Abbreviations

18F-FDG-PET: 18F-fluorodeoxyglucose positron emission tomography
3D: three-dimensional
AE: alveolar echinococcosis
BMZ: benzimidazoles
CEUS: Contrast enhanced ultrasound
CT: computed tomography
EMUC: Echinococcus multilocularis Ulm Classification
EMUC-CT: Echinococcus multilocularis Ulm Classification – Computed tomography
EMUC-US: Echinococcus multilocularis Ulm Classification – Ultrasound
E. multilocularis: Echinococcus multilocularis
et al.: et alii/et aliae/et alia
FDG: Fluorodeoxyglucose
H: Kruskal-Wallis H statistic
Max: Maximum
MBq: Megabecquerel
Min: Minimum
MRI: Magnetic Resonance Imaging
MSV: Millisievert
mm: Millimetre
n: sample size
p: p value
PET: Positron emission tomography
R0: R0-Resection – histopathologically confirmed complete removal of tumor tissue
SD: Standard deviation
SUV: standardized uptake value
SUVmax: maximum standardized uptake value
SUVmean: mean standardized uptake value
SUV _TLR_: standardized uptake value – tumor to liver ratio; tumor SUVmax/liver SUVmean
TOF: time of flight
US: B-scan ultrasound/ultrasonography
VOI: volume of interest
VPU: ViewPoint Ultrasound
WHO: World Health Organisation
